# Quinine in Cholera Infantum

**Published:** 1853-05

**Authors:** G. W. Booth

**Affiliations:** Carrollville, Miss.


Art. VI.—*Quinine in Cholera Infantum.* By G. W. Booth, M. D., of Carrollville, Miss.

I wrote a short and very imperfect article on Cholera Infantum which was published in the New York Medical Gazette in 1851. I therein gave my views of the etiology of this disease and my mode of treating it. I was in hopes that others would test the plan of treatment recommended and give the result of their experience to the profession. Presuming that no practitioner has deemed it necessary to adopt the plan then urged, as having been remarkably successful in my hands, and endorsed in some degree by Prof. L. P. Yandell, of Louisville, I have thought that it may not be amiss to call the attention of the profession to the subject again.

In the article referred to, I stated it as my opinion that the predisposing causes of Cholera Infantum and our autumnal fevers were identical, and that I had found the same mode of treatment successful in both forms of the disease, with variations to most local complications. My mode of treating the disease is with the disulphate of quinine, and I can truly say that in my hands it has been remarkably successful. I am confident that no person who will give the quinine a fair trial in this disease will feel disposed to lay it aside for any other mode of treatment at present known to the profession. If this brief article should have the effect of inducing others to adopt the quinine mode of treating this scourge of infancy in the Southern and South-Western portions of our country, I shall have effected all I intended by this short and imperfect communication.

May, 1853.

